# *In-Vitro* effect of Ficus deltoidea on the contraction of isolated rat’s uteri is mediated via multiple receptors binding and is dependent on extracellular calcium

**DOI:** 10.1186/1472-6882-13-359

**Published:** 2013-12-14

**Authors:** Naguib Salleh, Vivi Noryati Ahmad

**Affiliations:** 1 Department of Physiology, Faculty of Medicine, University of Malaya, Lembah Pantai, Kuala Lumpur 50603, Malaysia

**Keywords:** Ficus deltoidea, Uterotonin receptors, Extracellular Ca^2+^

## Abstract

**Background:**

Ficus deltoidea, is a perennial herb that is used to assist labor, firm the uterus post-delivery and to prevent postpartum bleeding. In view of its claimed uterotonic action, the mechanisms underlying plant’s effect on uterine contraction were investigated.

**Methods:**

Adult female SD rats were injected with 2 mg/kg 17β-oestradiol (E_2_) to synchronize their oestrous cycle. A day after injection, uteri were removed for *in-vitro* contraction studies. The dose dependent effect of Ficus deltoidea aqeous extract (FDA) on the tension produced by the isolated rat’s uteri was determined. The effects of atropine (2×10^-8^ M), atosiban (0.5 IU), THG113.31 (10 μM), oxodipine (0.25 mM), EDTA (1 mM), 2-amino-ethoxy-diphenylborate (2-APB) (40 mM) and thapsigargin (1 mM) on the maximum force of contraction (Emax) achieved following 2 mg/ml FDA administration were also investigated.

**Results:**

FDA induced *in-vitro* contraction of the isolated rat’s uteri in a dose-dependent manner. Administration of atropine, atosiban and THG113.31 reduced the Emax with atosiban having the greatest effect. The Emax was also reduced following oxodipine and EDTA administration. There was no significant change observed following 2-APB administration. Thapsigargin, however, augmented Emax.

**Conclusions:**

FDA-induced contraction of the isolated rat’s uteri is mediated via multiple uterotonin receptors (muscarinic, oxytocin and prostaglandin F2α) and was dependent on the extracellular Ca^2+^. Contraction, however, was not dependent on the Ca^2+^ release from the internal stores. This *in-vitro* study provides the first scientific evidence on the claimed effect of Ficus Deltoidea on uterine contraction.

## Background

Uterotonic plants, are plants that stimulate uterine contraction and have been used since the ancient times to assist labor, remove the retained placenta, treat post partum bleeding and as an abortifacient [1]. Despite their use, scientific evidence to substantiate their beneficial effects are still being explored Several plant extracts have been reported to induce uterine contraction which include the leaves extract of P. nigrescens, C. bonduc and A. africanus [2-4], roots extract of C. papaya [5,6] and L. pumila [7] and several other extracts from various plant species [8,9].

One of the plants that is popularly used among the Southeast Asian women to induce uterine contraction is Ficus deltoidea*,* which belongs to the family Moraceae [10]. Ficus deltoidea has been claimed to possess uterotonic properties that can assist labor as well as can promote involution of the uterus, cervix and vagina during the postpartum period. In addition, it has also been used to treat abnormalities in the menstrual cycle and for birth spacing purposes. Ficus deltoidea is a small perennial herb which rarely exceeds 2 meters in height and is domestically cultivated. It is known by various names such as Mas Cotek in Malaysia, Tabat Barito in Indonesia, Agoluran in The Philippines and Kangkalibang in Africa [11]. Different sub-species can be identified based on the leaf shape; with the female plants possessing larger and more round leaves, whereas the males are smaller with long and round leaves [12]. All parts of this plant are medicinally useful. The leaves (when boiled), stems, roots and fruits are traditionally use to treat arthritis, boost the immune system and increase the sexual desire [13]. Additionally, the leaves are also claimed to be a powerful fat burner, help to decrease plasma cholesterol level and are experimentally proven to lower the blood glucose level [14]. Recently, the fruit extract of Ficus deltoidea was found to possess antidiabetic and antioxidant properties [15].

The notion that Ficus deltoidea affects uterine contraction was based on several previous studies involving other Ficus species which also indicate their effect on the uterine contractility. Most of these studies were conducted *in-vitro* using isolated uteri from rodents. Bafor et al. [16] conducted an *in-vitro* study using isolated strips of mouse uteri to investigate the effect of the active constituent of Ficus exasperata leaf extract on uterine contraction. They have found that metabolites generated from this extract possess both tocolytic and uterotonic activities. Earlier, *in-vitro* contraction studies using isolated rat’s uteri revealed both stimulatory [17] and inhibitory [18] effects of the unpurified extract of Ficus exasperata. Further to this, Watcho et al., [1] demonstrated that fruit extract of Ficus asperifolia stimulates *in-vitro* contraction of the uteri isolated from oestrogenized rats. Another Ficus species, Ficus capensis was shown to inhibit *in-vitro* contraction also in the isolated rat’s uteri [19]. In view of the fact that Ficus species was found to affect uterine contractility, we hypothesized that Ficus deltoidea, which is also from the Moraceae family, may affect uterine contraction as traditionally claimed. Therefore, using similar experimental model [1,17,18,20], we investigated Ficus Deltoidea effect on uterine contraction.

## Methods

### Preparation of *F. Deltoidea* Aqeous extract (FDA)

Leaves of *F deltoidea* from female sub-species used in this study were supplied by Delto Medicama Plantation, Kuala Selangor, Malaysia. The plant sample was deposited at the Herbarium in Rimba Ilmu, University of Malaya, Kuala Lumpur for authentication and identification purposes with Herbarium number: KLU 46469. The leaves were air-dried, cut into small pieces and grounded into powder form. Each of the pulverized parts were weighed (100 grams) and boiled twice in 1 litre distilled water for 4 hours. The aqueous extract was then concentrated by heating at 60°C and was later subjected to freeze- drying (yield 7.36% and 11.61% w/w, dry weight basis for leaf) and was stored in a container until further use. Stock solution was obtained by dissolving small aliquots of this extract in water.

### Uterine tissue preparation and *In-Vitro* contraction study

Adult female Wistar Kyoto (WKY) rats weighing 250 grams were purchased from the Animal House, Faculty of Medicine, University of Malaya, Kuala Lumpur. The rats were housed in a controlled environment with temperature kept at 25°C, relative humidity between 30–70%, 12 hours light–dark cycle and had free access to rodent food pellet and tap water *ad libitum*. Cleanliness of the housing environment was maintained daily. Shredded recycled paper was used as bedding. Each group consists of six rats (n = 6). All experimental procedures were approved by the University of Malaya Medical Center Animal Ethics Committee (Ethics Reference No: FIS/ 01/12/2008/ NS (R)). Intact, non-ovariectomised female WKY rats were treated with high dose of E_2_ at 2 mg/kg/day to synchronize their oestrous cycle [17]. A day after injection, the rats were humanely sacrificed and the uteri were immediately removed and placed into a physiological solution. The tissue was then placed vertically in an organ bath containing solution with the following electrolytes composition: NaCl (155 mM), KCl (4.5 mM), MgCl_2_ (1.0 mM), CaCl_2_ (2.0 mM) and D- glucose (10 mM) while the pH was maintain at 7.40 with NaOH. The temperature of the organ bath was maintained at 37°C. 95% O_2_ and 5% CO_2_ was continuously delivered into the bathing solution. Each uterine strip was placed under optimum resting force of 1 g and was allowed to equilibrate for 30 minutes prior to drug administration. During this period, the strips were washed with 10 ml fresh physiological solution every 15 minutes according to the method by Oropeza et al., [21]. Each experiment was repeated six times using new uterine strips from different rats (n = 6). Contractile forces were recorded isometrically by a force transducer which was connected to a bridge amplifier and to the PowerLab data acquisition system (ADI Instrument, Australia).

FDA was added in a dose-dependent manner (0.125-4.0 mg/ml) and the dose- response effect was then recorded. Preliminary investigation revealed that 1 × 10^-2^ M Ach (Sigma-Aldrich), 7 I.U. oxytocin (Sigma-Aldrich) and 5 μg/ml PGF2α (Sigma-Aldrich) produced maximum force of contraction (Emax), which values differ between the respective agonists. Meanwhile, 2 mg/ml FDA also resulted in maximum contraction (Emax), however with a lower Emax than other tested agonists. Atropine (Sigma-Aldrich) (2 × 10^-8^ M), a muscarinic receptor antagonist; atosiban (Fluka Co) (0.5 IU), an oxytocin receptor antagonist, THG113.31 (Theratechnologies) (10 μg/ml), a PGF2α receptor antagonist, oxodipine (Sigma-Aldrich) (0.25 μM), an L-type Ca^2+^-channel blocker, 2-APB (Sigma Aldrich) (40 μM), an IP_3_ receptor (IP_3_R) blocker, thapsigargin (Sigma Aldrich) (1 μM), a sarcoplasmic reticulum Ca^2+^-ATPase (SERCA) inhibitor and EDTA (Sigma Aldrich) (1 mM), a Ca^2+^ chelator were administered to investigate the mechanism underlying FDA effect on uterine contraction. In order to observe the effect of these inhibitors, 2 mg/ml FDA was initially added into the bathing solution and once contraction was stable at Emax, these inhibitors were either individually or simultaneously added and their effects on the Emax were then recorded.

### Statistical analysis

Results were expressed as mean ± SEM. Data was analyzed using Student’s *t*- test. p < 0.05 was considered to be statistically significant.

## Results

### Dose-dependent effect of FDA on uterine contraction

**Figure 1:**
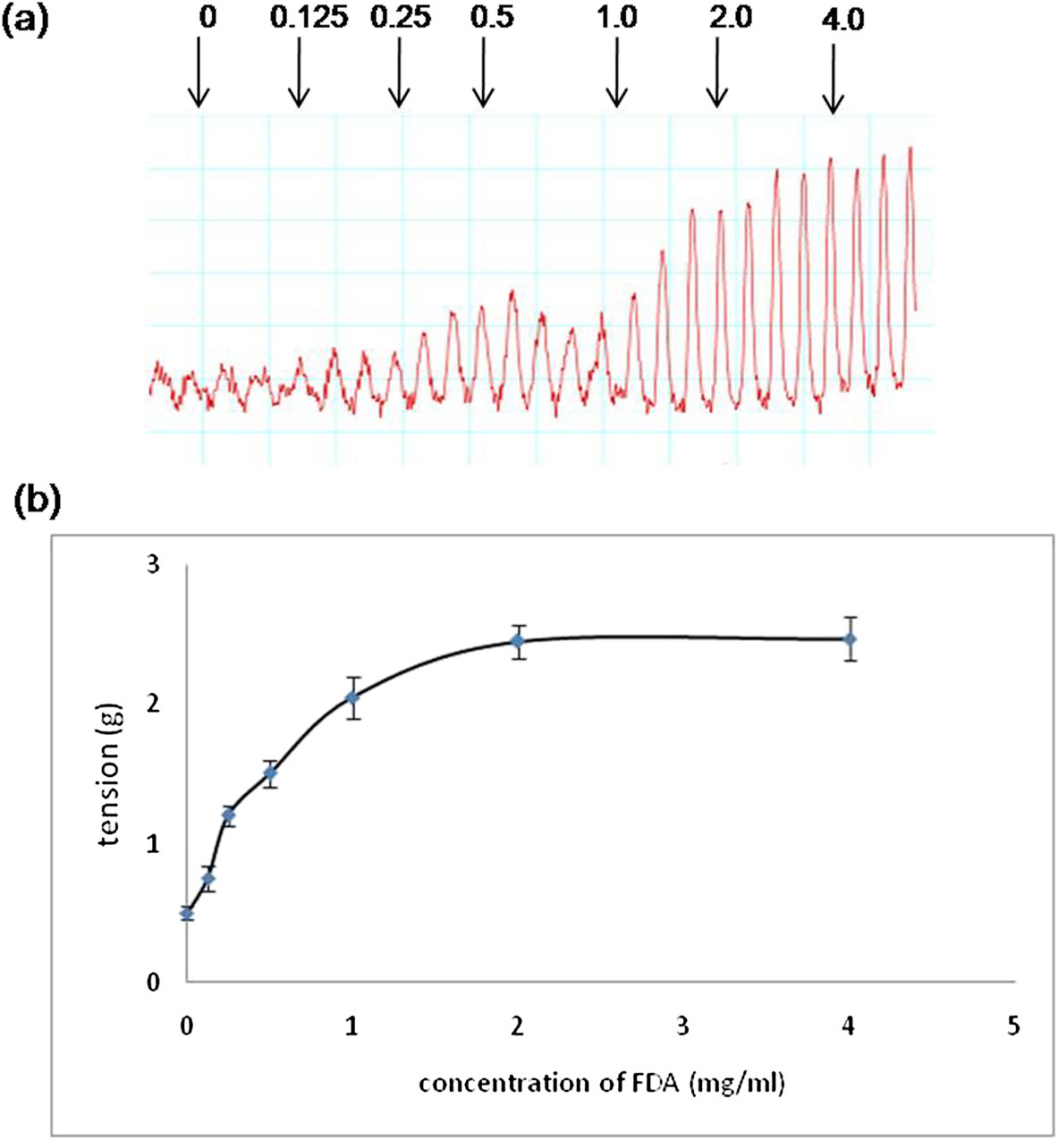
**The effect of FDA on uterine contraction. (a)** Tracing of isometric uterine contraction following administration of various doses of FDA and **(b)** mean tension generated from six isolated uterine horns obtained from different oestrogenized rats, which were exposed to various doses of FDA at concentrations ranging between 0.125 to 4 mg/ml. There was a dose-dependent increase in the tension with increasing doses of FDA. The tension recorded following administration of 2 mg/ml FDA was 4.9 times higher than the control. n = 6 (* p < 0.05 as compared to control).

In Figure 1, the force of contraction increases with increasing doses of FDA. In the control group, the force recorded was 0.5 ± 0.05 g tension, which was the baseline contraction in oestrogenized rats’ uteri. At 0.25 mg/ml, the force generated was 2.4 times greater than the control. Meanwhile, the forces increase by 3.0, 4.1 and 4.9 times following administration of 0.5, 1 and 2 mg/ml FDA respectively with 2 mg/ml FDA produced the maximum tension (Emax).

### Effect of atropine, THG113.31 and atosiban on the Emax induced by 2 mg/ml FDA

**Figure 2:**
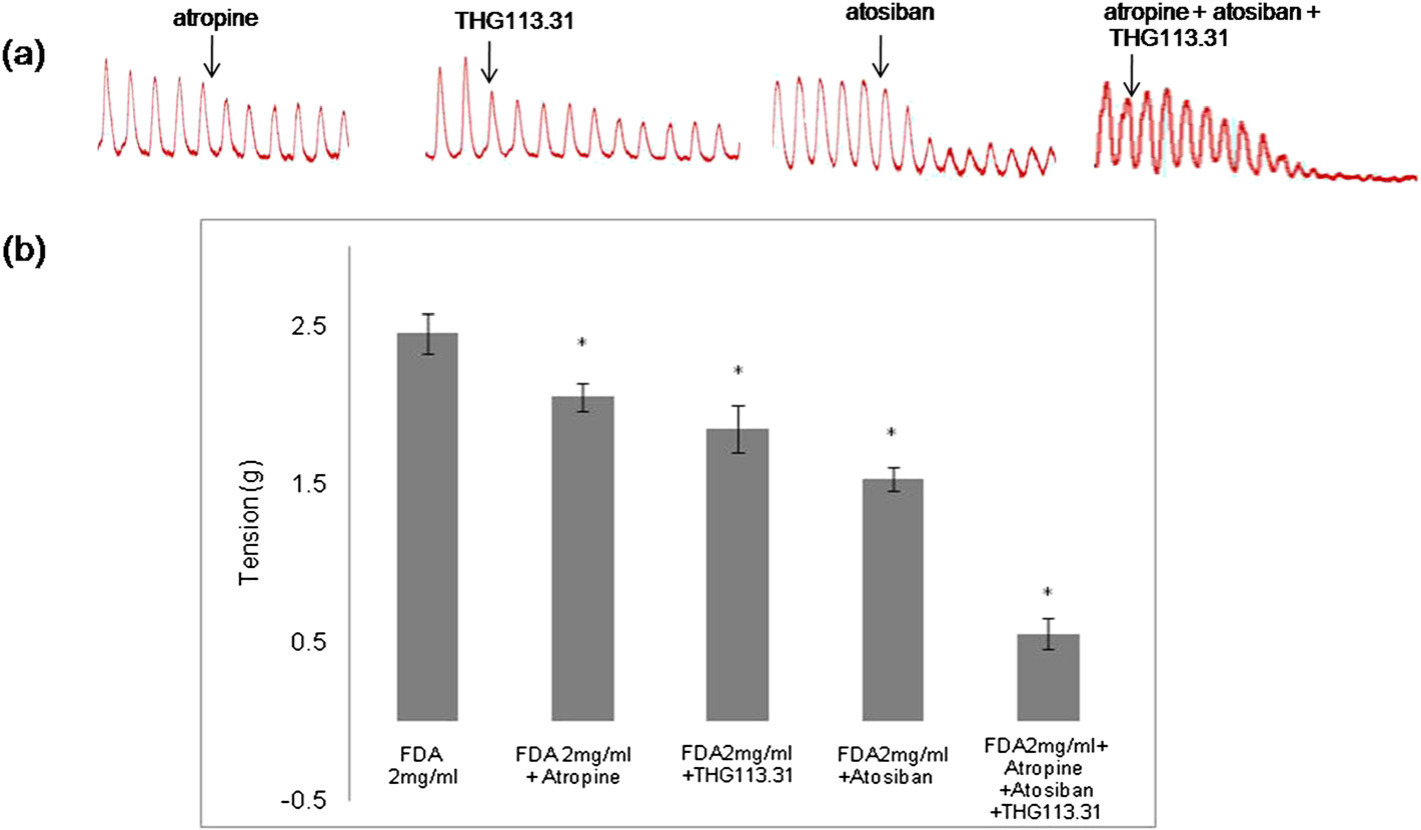
**The effect of selected receptor antagonists on FDA-induced uterine contraction. (a)** Representative tracings of isometric uterine contraction following FDA administration in the presence of various antagonists and **(b)** mean Emax following administration of FDA at 2 mg/ml and in the presence of atropine, THG113.31 and atosiban. Atropine caused the least inhibition while atosiban produced the greatest inhibition on the Emax. Concomitant administration of all three antagonists resulted in a remarkable decrease in the Emax. n = 6 rats per group, *p < 0.05.

In Figure 2, administration of muscarinic receptor antagonist, atropine, into the bathing solution containing isolated uterine tissue pre-exposed to 2 mg/ml FDA resulted in the Emax to decrease by 1.19 times. Meanwhile, administration of THG113.31, a non-competitive inhibitor for PGF2α receptor, as well as atosiban, an oxytocin receptor blocker resulted in the Emax to also decrease by 1.32 and1.60 times respectively. Simultaneous administration of atropine, atosiban and THG113.31 resulted in 4.45 times decrease in Emax as compared to 2 mg/ml FDA administration alone.

### Relative potency of FDA as uterotonin

In Table 1, the relative potency of FDA was compared to other uterotonins. The Emax produced following administration of 2 mg/ml FDA was 2.45 ± 0.10 g. Meanwhile, the Emax produced following administration of 1 × 10^-2^ M Ach, 7 I.U oxytocin and 5 μg/ml PGF2α were 2.98 ± 0.25, 3.51 ± 0.47 and 3.43 ± 0.19 g respectively.

**Table 1:** Relative potency of FDA as compared to other uterotonins

	**Tension (g)**
FDA	2.45 ± 0.10
Acetylcoline (Ach)	2.98 ± 0.25
Oxytocin	3.51 ± 0.27
Prostaglandin F2α (PGF2α)	3.43 ± 0.19

FDA is 1.43 times less potent than oxytocin. This extract is also 1.40 and 1.21 times less potent than PGF2α and Ach respectively. n = 6 per treatment group.

### Effect of oxodipine and EDTA on the Emax induced by 2 mg/ml FDA

**Figure 3:**
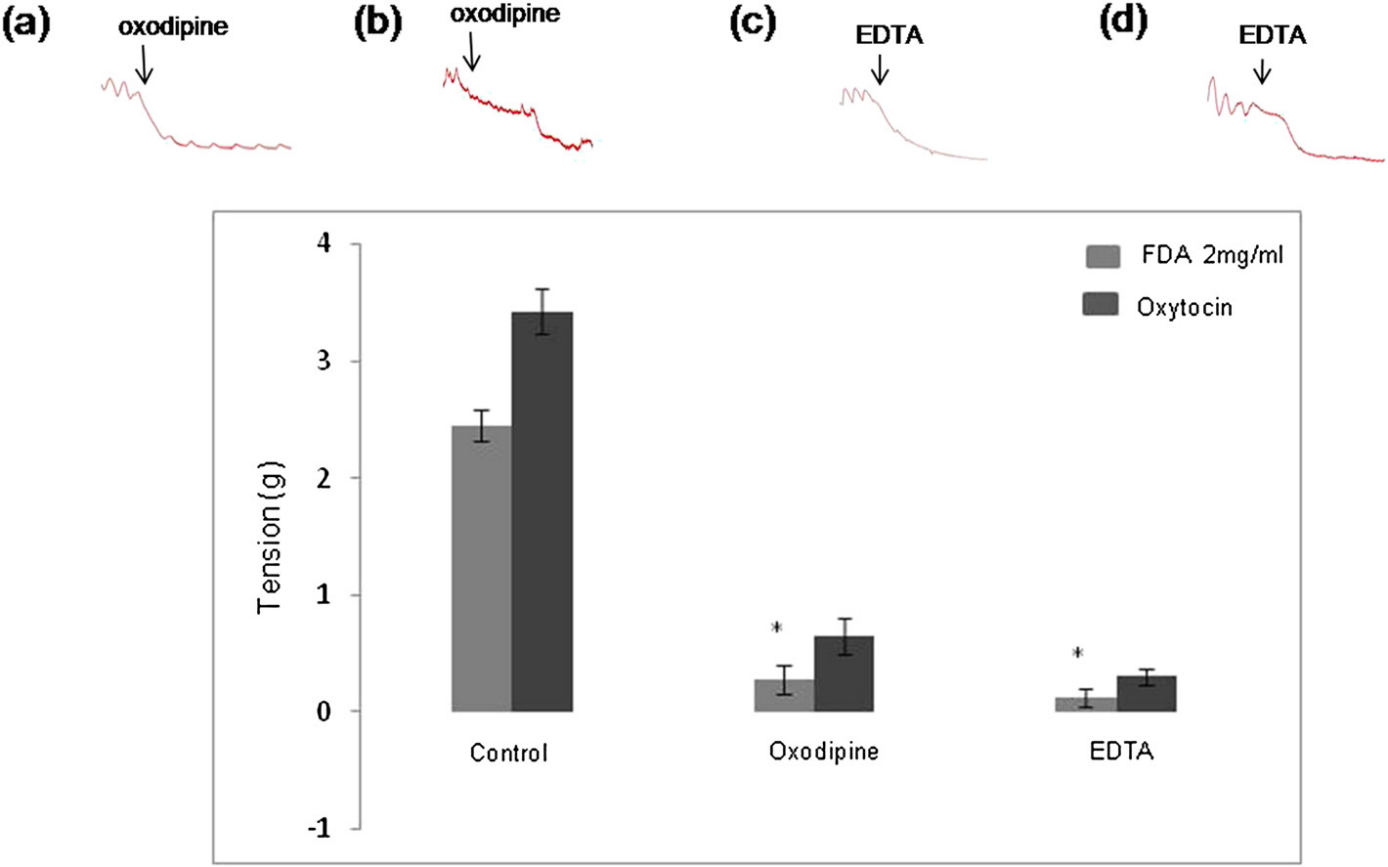
**The effect of calcium channel blocker and extracellular calcium removal on FDA-induced uterine contraction.** Representative image of isometric uterine contraction following administration of **(a)** 2 mg/ml FDA plus oxodipine, **(b)** oxytocin plus oxodipine, **(c)** 2 mg/ml FDA plus EDTA and **(d)** oxytocin plus EDTA while bar chart shows the Emax following oxodipine and EDTA administration as compared to the control. Contraction was almost abolished following EDTA administration in the isolated uteri pre-exposed to FDA. n = 6 (* p < 0.05).

In Figure 3, administration of oxodipine, a voltage-gated L-type Ca^2+^ channel antagonist into the bathing solution containing isolated uterine tissue pre-exposed to 2 mg/ml FDA resulted in the Emax to decrease by 88.5%. Meanwhile, administration of EDTA into this solution which resulted in depletion of extracellular Ca^2+^ caused the Emax to decrease by a greater percentage (96.8%). Lesser degree of inhibition by oxodipine and EDTA in isolated uterine tissue pre-exposed to oxytocin indicated that this effect of oxytocin was not solely dependent on the extracellular Ca^2+^.

### Effect of 2-APB and thapsigargin on the Emax induced by 2 mg/ml FDA

**Figure 4:**
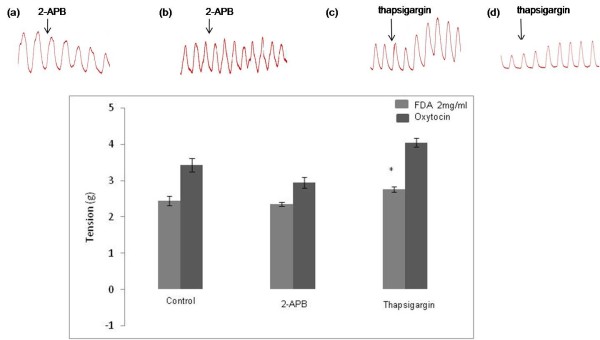
**The effect of IP3R blocker and SERCA inhibitor on FDA-induced uterine contraction.** Representative image of isometric uterine contraction following administration of **(a)** oxytocin plus 2-APB, **(b)** FDA 2 mg/ml plus 2-APB, **(c)** oxytocin plus thapsigargin and **(d)** FDA 2 mg/ml plus thapsigargin while bar chart shows the Emax following oxytocin and FDA administration with and without the presence of 2-APB and thapsigargin. No significant difference was noted following 2-APB administration in the FDA treated group while thapsigargin caused a slight but significant increase in the Emax.

In Figure 4, administration of 2-APB, an IP3R blocker into the bathing solution containing isolated uterine tissue pre-exposed to 2 mg/ml FDA did not cause any significant changes in the Emax produced. Meanwhile, administration of SERCA inhibitor, thapsigargin, resulted in 8.5% increase in the Emax as compared to FDA alone. 2-APB caused a significant decrease in the Emax in isolated uterine tissue pre-exposed to oxytocin, while thapsigargin administration resulted in the opposite effect*.*

## Discussion

To the best of our knowledge, this study is the first to display uterotonic effect of Ficus deltoidea, which justifies the claim that this plant assists in uterine contraction. We have shown that FDA effect is mediated via muscarinic, oxytocin and PGF2α receptors and is dependent on the extracellular Ca^2+^. These mechanisms were confirmed from inhibition of the maximum tension (Emax) produced by 2 mg/ml FDA following administration of the antagonists to these receptors and inhibitors to the Ca^2+^ channels. FDA is 1.43 times less potent than oxytocin, which is a gold standard uterotonin [15]. Apart from Ficus deltoidea, a few other Ficus species including Ficus exasperata [17,22] and Ficus asperifolia [1] were also reported to stimulate uterine contraction, suggesting that uterotonic effect is common to the Ficus species.

Our findings suggested that FDA-induced uterine contraction was mediated mainly via the oxytocin receptor as evidenced by the highest degree of inhibition of the Emax by atosiban. Moderate inhibition of the Emax by THG113.31 suggested that FDA binding to PGF2α receptor produced moderate degree of contraction while the lowest inhibition by atropine suggested that FDA binding to the muscarinic receptor produced the least degree of contraction. The cumulative inhibitory effect observed following concomitant administration of atropine, THG113.31 and atosiban confirmed the involvement of all three receptors in mediating FDA-induced uterine contraction. The presence of muscarinic, oxytocin and PGF2α receptors in the uterus has been previously reported [23-25]. These receptors were reported to be up-regulated by E_2_ and in the late pregnancy particularly at term [26]. In view of this, high dose E_2_ administration to the rats prior to the experiment can result in an increase in the number of these uterotonin receptors, potentiating the effect of FDA on uterine contraction.

There is a possibility that the greatest effect produced following FDA binding to the oxytocin receptor (as evidenced by the greatest inhibition of the Emax by atosiban) was due to high number of this receptor expression in the uterus. Meanwhile, lesser inhibition by THG113.31 and atropine suggested that the number of PGF2α and muscarinic receptors expressed was lower than the number of oxytocin receptor expression. Up-regulation of oxytocin receptor by E_2_ and at term [26,27] has been reported in human [28], rat [25] and mouse [29] uterus while muscarinic and PGF2α receptors expression has also been reported in rat [30-33], rabbit [34] and human [23] uterus which were also being up-regulated by E_2_[24]. Previous reports also indicate that oxytocin receptor expression in the uterus is the highest [35,36], supporting our observation that FDA effect was mostly mediated via this receptor binding. Apart from the increase in the number of receptors, high affinity FDA binding to the oxytocin receptor may also result in the observed effect. Oxytocin and PGF2α have been reported to play an important role in the myometrial contraction [37]. Oxytocin-induced myometrial contraction has been shown in estrogen-primed non-pregnant swine uteri [38,39]. Activation of the oxytocin and PGF2α receptors which are coupled to G protein alpha(q) stimulates uterine contraction through activating the phospholipase C/Ca^2+^ dependent pathway, while activation of the muscarinic receptor which is coupled to G protein alpha(i) potentiates contraction through inhibiting the cAMP production [40]. Besides Ficus deltoidea, other Ficus species including Ficus asperifolia has also been reported to induce uterine contraction through binding to the muscarinic, oxytocin and histamine receptors in the uterus [41].

Ca^2+^, which is essential for smooth muscle contraction [42], can be derived from the intracellular stores and/or extracellular fluid [42,43]. Extracellular Ca^2+^ enters the cell via the voltage-gated dihydropyridine channels at the myocyte plasma membrane [44]. Following the opening of this channel, Ca^2+^ enters down its concentration gradient. This will then trigger the release of Ca^2+^ from the intracellular stores [45]. In this study, the involvement of intracellular and extracellular Ca^2+^ in myometrial contraction was investigated following oxytocin and 2 mg/ml FDA administration. Our findings indicate that oxytocin-induced uterine contraction depends mostly on the extracellular Ca^2+^ while intracellular Ca^2+^ is also required for contraction. Following binding of oxytocin to its G protein-coupled receptor, phospholipase C (PLC) will be activated which causes an increase in inositol trisphosphate (IP3) and diacylglycerol (DAG) levels. IP3 activates the IP3R receptor at the sarcoplasmic reticulum membrane which causes the release of stored Ca^2+^ into the cytosol. Increased cytosolic Ca^2+^ will further induced extracellular Ca^2+^ influx [42], resulting in a further rise in the intracellular Ca^2+^ level. Ca^2+^ will then binds to calmodulin, which activates the myosin light chain kinase leading to phosphorylation of myosin light chains, triggering contraction [46].

A marked decrease in the Emax following oxodipine and EDTA administration suggested the dependency of FDA-induced uterine contraction on the extracellular Ca^2+^. This could be similar to the contraction induced by wild ginger (Costus speciosus) rhizome [47] and pomegranate (Punica granatum L., Punicaceae) seed [48] extracts which was also shown to solely depend on the extracellular Ca^2+^. In this study, FDA binding to the muscarinic, oxytocin and PGF2α receptors may trigger the extracellular Ca^2+^ influx prior to contraction. Although FDA has been shown to mediate its uterotonic effect, mostly via oxytocin receptor binding, the contraction produced however does not depend on the intracellular Ca^2+^ as evident from the lack of inhibition on the Emax by 2-APB. This is in contrast to oxytocin-induced uterine contraction, whereby its dependency on the intracellular Ca^2+^ was evidenced from the inhibition of Emax by 2-APB. We speculated that the inability of FDA to induce the release of Ca^2+^ from the internal stores could be due to its inability to provide adequate stimulus to trigger the intracellular cascade leading to the release of Ca^2+^ from the intracellular stores, despite of its binding to the oxytocin receptor. On the other hand, FDA may also bind at lower affinity to other uterotonin receptors, which may explain lesser potency of FDA as uterotonin as compared to oxytocin, PGF2α and Ach.

In addition to the binding to the oxytocin receptor, FDA-induced extracellular Ca^2+^ influx could also involve other agonists’ receptor binding. This includes the PGF2α receptor; which was found to mediate uterine contraction in the laying hens via inducing the influx of extracellular Ca^2+^[49]. Our finding has shown that administration of thapsigargin, a SERCA inhibitor resulted in a slight but significant increase in the Emax induced by oxytocin and FDA. This effect could be due to the depletion of stored Ca^2+^ by thapsigargin which inhibit the reuptake of cytosolic Ca^2+^ into the sarcoplasmic reticulum. The consistently high cytosolic Ca^2+^ will activate extracellular Ca^2+^ entry which would further enhance uterine smooth muscle contraction [50].

## Conclusion

Using *in-vitro* model, our study has provided the first scientific evidence to support the claim that Ficus deltoidea stimulates uterine contraction. The active compound that is responsible in mediating this effect is currently unknown, although Ficus deltoidea has been reported to contain flavonoids isovitexin, vitexin [51], proantrocyanidins, flavan-3-ol monomer and flavones glycosides [52]. Phytochemical analyses further revealed the presence of tannins, tripterpenoids and phenols although alkaloids and steroids were not commonly found [10]. This *in-vitro* study using isolated rodent’s uteri therefore provides preliminary evidence which could be used to further explore the *in-vivo* effect of this plant compound on uterine contraction.

## Competing interests

Authors have nothing to disclose.

## Authors’ contributions

NS is the project leader who planned this study, was involved in data interpretation and prepared the manuscript for publication. Meanwhile, VNA conducted all experiments. Both authors have read and approved the manuscript.

## Pre-publication history

The pre-publication history for this paper can be accessed here:

http://www.biomedcentral.com/1472-6882/13/359/prepub
